# Biofilm-Associated Antibiotic Resistance in Indian Clinical Isolates of Klebsiella pneumoniae: A Systematic Review With a Post Hoc Meta-Analysis of blaNDM Prevalence

**DOI:** 10.7759/cureus.105056

**Published:** 2026-03-11

**Authors:** Yatindra Kumar, Soni Narayan, Sasikala Kathiresan, M Rose John, K Jnapika, Lalitha Soumya Johnson, Shouvik Mitra, Nabina Nazeer, Mukul Singh, Akshay VP, Delna NS, Renu Mishra, Roshni Pal, Shubhrit Shrivastava

**Affiliations:** 1 Pharmacology and Therapeutics, Chhatrapati Shivaji Maharaj University, Panvel, IND; 2 General Medicine, Manipal - TATA Medical College, Jamshedpur, IND; 3 Obstetrics and Gynaecology, All India Institute of Medical Sciences, Madurai, Madurai, IND; 4 Emergency Medicine, Sree Narayana Institute of Medical Sciences, Ernakulam, IND; 5 Emergency Medicine, Gayatri Vidya Parishad Institute of Healthcare and Medical Technology, Visakhapatnam, IND; 6 Biotechnology and Microbiology, Dr. Janaki Ammal Campus, Kannur, IND; 7 Biotechnology, Kurukshetra University, Kurukshetra, IND; 8 Emergency Medicine, General Hospital - Thiruvananthapuram, Thiruvananthapuram, IND; 9 Surgery, All India Institute of Medical Sciences, Gorakhpur, Gorakhpur, IND; 10 Biotechnology, Mansarovar Global University, Bhopal, IND; 11 Molecular Biology, BioDesk INDIA Labs, Bhopal, IND; 12 Allied Health Sciences, Al-Azhar Medical College and Super Specialty Hospital, Thodupuzha, IND; 13 Microbiology, Sri Sathya Sai College for Women, Bhopal, IND; 14 Microbiology, Career College Bhopal, Bhopal, IND; 15 Biochemistry, BioDesk INDIA Labs, Bhopal, IND

**Keywords:** antimicrobial resistance, bacterial virulence, biofilm formation, carbapenem resistance, hospital-acquired infections, klebsiella pneumoniae, multidrug resistance, nosocomial pathogens, resistance patterns, systematic review

## Abstract

This review aimed to systematically examine biofilm production and antibiotic resistance in *Klebsiella pneumoniae* isolates from Indian health care settings. An extensive literature search was conducted across scholarly databases for studies published in the past decade reporting biofilm formation and carbapenem resistance in Indian isolates of *K. pneumoniae*. Studies were screened, and data were extracted from eligible studies.

Ten studies met the inclusion criteria, representing diverse Indian clinical settings. For the association between biofilm formation and carbapenem resistance, only two studies met the criteria for quantitative pooling. Due to the very small number of studies, substantial methodological differences, and wide variability in effect estimates, a formal meta-analysis was not performed. Instead, a qualitative synthesis was conducted, which indicated a trend toward higher carbapenem resistance among strong biofilm producers. Separately, a post hoc meta-analysis was performed for *bla*NDM prevalence. Four studies contributed data to this analysis, yielding a pooled prevalence of 43% (95% CI: 30%-58%). Across studies, resistance genes were identified for 12 antibiotic classes, including carbapenemases (*bla*NDM and *bla*OXA-48), extended-spectrum β-lactamases (ESBLs) (*bla*CTX-M and *bla*TEM), aminoglycoside-modifying enzymes (*rmtB* and *armA*), and efflux pump regulators.

Our results suggest an association between strong biofilm formation and carbapenem resistance in Indian *K. pneumoniae* isolates. The widespread presence of resistance genes across multiple antibiotic classes underscores the urgent need for surveillance and targeted infection-control strategies. The high prevalence of *bla*NDM among Indian clinical isolates highlights the clinical threat posed by these organisms in India.

## Introduction and background

Bacterial infections remain a major cause of morbidity and mortality worldwide, and their impact has been further intensified by the rapid emergence of antimicrobial resistance (AMR). Recent global estimates indicate that AMR was associated with approximately 4.95 million deaths worldwide in 2019, with 1.27 million deaths directly attributable to bacterial AMR, highlighting its substantial public health burden [1]. Among the most concerning pathogens contributing to this crisis is *Klebsiella pneumoniae*, a Gram-negative, encapsulated bacillus belonging to the family *Enterobacteriaceae* [1,2].

The management of *K. pneumoniae* infections has become increasingly challenging due to the pathogen’s capacity to acquire and express multiple antibiotic resistance mechanisms, making it a prominent member of the ESKAPE group of multidrug-resistant (MDR) organisms (ESKAPE refers to a group of six highly virulent and antibiotic-resistant bacterial pathogens: *Enterococcus faecium*, *Staphylococcus aureus*, *Klebsiella pneumoniae*, *Acinetobacter baumannii*, *Pseudomonas aeruginosa*, and *Enterobacter* species) [2].

One of the key virulence factors contributing to the persistence and resistance of *K. pneumoniae* in clinical settings is its ability to form biofilms. Biofilms are structured communities of bacterial cells embedded in a self-produced matrix of extracellular polymeric substances (EPS), which adhere to biotic or abiotic surfaces [3]. This mode of growth provides significant protection against host immune responses and antibiotic treatment, often leading to chronic or recurrent infections. Within a biofilm, *K. pneumoniae* exhibits altered gene expression, metabolic dormancy, and restricted antibiotic penetration, collectively contributing to elevated minimum inhibitory concentrations (MICs) and phenotypic resistance. Importantly, biofilm formation facilitates the horizontal gene transfer of resistance elements, such as plasmid-encoded beta-lactamases and carbapenemases (e.g., *bla*KPC and *bla*NDM), amplifying the resistance burden [3,4].

Carbapenems represent one of the most important classes of β-lactam antibiotics and are often considered the last-line therapeutic option for the treatment of severe infections caused by MDR Gram-negative bacteria. The emergence of carbapenem-resistant *K. pneumoniae* (CRKP) has therefore become a major global concern, as resistance to these agents severely limits available treatment options and is frequently associated with poor clinical outcomes, including increased mortality, prolonged hospitalization, and higher healthcare costs. Carbapenem resistance in *K. pneumoniae* is primarily mediated through the production of carbapenemase enzymes, such as KPC, NDM, OXA-48, and VIM, which are often carried on mobile genetic elements that facilitate rapid dissemination among bacterial populations [1,3].

In India, the situation is particularly concerning due to the high rates of hospital-acquired infections and widespread antibiotic misuse, which accelerate the emergence and dissemination of MDR and extensively drug-resistant (XDR) strains [5]. Recent reports suggest a rising prevalence of biofilm-producing *K. pneumoniae* isolates in Indian hospitals, with high resistance rates to critical antibiotics, such as carbapenems, aminoglycosides, and fluoroquinolones. These biofilm-forming strains not only complicate infection control efforts but also compromise clinical outcomes, including prolonged hospital stays, increased healthcare costs, and higher mortality rates [5,6].

The current systematic review aims to synthesize observational evidence on the prevalence, antibiotic resistance profiles, and clinical associations of *K. pneumoniae* isolated from human clinical samples in India over the past decade. By focusing exclusively on Indian populations and clinical settings, the study will generate context-specific insights that can inform antimicrobial stewardship policies, guide empiric therapy choices, and shape future research directions. This systematic review is attempted in light of the World Health Organization’s (WHO) designation of CRKP as a critical-priority pathogen for research and drug development [7]. 

## Review

Materials and methods

This systematic review was conducted as per the protocol registered with the International Prospective Register of Systematic Reviews (PROSPERO; CRD42024621381), and the public version of the full protocol - including the detailed search strategy, data extraction templates, risk of bias forms, and other supplementary materials (Appendix 1) - is available on the Open Science Framework (OSF) (https://doi.org/10.17605/OSF.IO/N5AYH) [8]. The review adhered to the PRISMA 2020 (Preferred Reporting Items for Systematic Reviews and Meta-Analyses) guidelines in reporting the findings [9].

Eligibility Criteria

The inclusion and exclusion criteria for this systematic review were developed using the PEO (Population, Exposure, Outcome) framework [9]. The population of interest comprised patients with confirmed *K. pneumoniae* infections in clinical settings across India. The primary exposure was *K. pneumoniae* infection, and the outcomes considered were the prevalence of biofilm production and associated antibiotic resistance profiles, especially carbapenem resistance, including resistance rates and resistance genes. Eligible studies were restricted to observational designs published in the English language between 2014 and 2025. Studies were included only if they reported either the prevalence of biofilm-producing *K. pneumoniae* or antibiotic resistance data specific to these isolates.

Studies were excluded if they were in vitro or animal experiments, reviews, editorials, or conference abstracts without full data. Additionally, studies were excluded if they lacked clear documentation of biofilm detection methods or antibiotic resistance outcomes, or if they were based on non-clinical samples or populations outside India.

Information Sources and Search Strategy 

Databases searched include, but are not limited to, PubMed, Scopus, and Google Scholar. The search was limited to studies published between 2014 and 2025 (the last search was conducted in March 2025) and written in English. An example PubMed search strategy was: (“Klebsiella pneumoniae” AND biofilm) AND (“antibiotic resistance” OR carbapenem OR blaNDM) AND (observational OR cross-sectional OR cohort) AND India. The complete database-specific search strings are provided in the OSF-registered protocol [8]. 

Study Selection

Titles and abstracts were independently screened by two reviewers. Full texts were retrieved for potentially eligible articles and assessed against the eligibility criteria. Discrepancies were resolved through consensus or adjudication by a third reviewer.* *

Data Extraction

Data were extracted independently by two reviewers using a standardized form, including author, year, region, study design, sample size, clinical setting, type of infection, biofilm production, and resistance patterns. Non-open-access studies were also included initially, and corresponding authors were contacted. Only those studies for which no response was received were excluded. Communication records and excluded papers can be found on OSF [8]. 

Risk of Bias Assessment

The methodological quality of the included studies was evaluated using the Newcastle-Ottawa Scale (NOS) for observational studies [10] and the Joanna Briggs Institute (JBI) Critical Appraisal Checklist for case reports [11]. Case reports were appraised separately and were not considered for quantitative synthesis. The quality assessment examined domains including sample representativeness, measurement validity, control of confounding variables, and appropriateness of statistical analysis. Based on the scoring criteria, studies were categorized as having low, moderate, or high risk of bias. Studies identified as having a high risk of bias were not excluded from the qualitative synthesis in order to preserve the comprehensiveness of the review; however, they were carefully considered during interpretation and were not included in quantitative analyses where applicable.

Data Synthesis and Statistical Analysis

Given the limited number of studies with directly comparable CRKP prevalence data across biofilm categories, a qualitative synthesis was conducted for this outcome instead of a planned meta-analysis. A post hoc meta-analysis was performed to estimate the pooled prevalence of the *bla*NDM gene among *K. pneumoniae* isolates, using a random-effects model (DerSimonian-Laird method) with logit transformation of proportions. Between-study heterogeneity was assessed using Cochran’s Q, τ², and I² statistics, and a 95% prediction interval was calculated. Statistical analyses were conducted in R software (version 4.4.2; R Foundation for Statistical Computing, Vienna, Austria), with codes adopted from "Meta-analysis With R" by Schwarzer et al. [12]. Resistance genes were further categorized by antibiotic class based on their molecular resistance mechanisms.

Results

**Figure 1:**
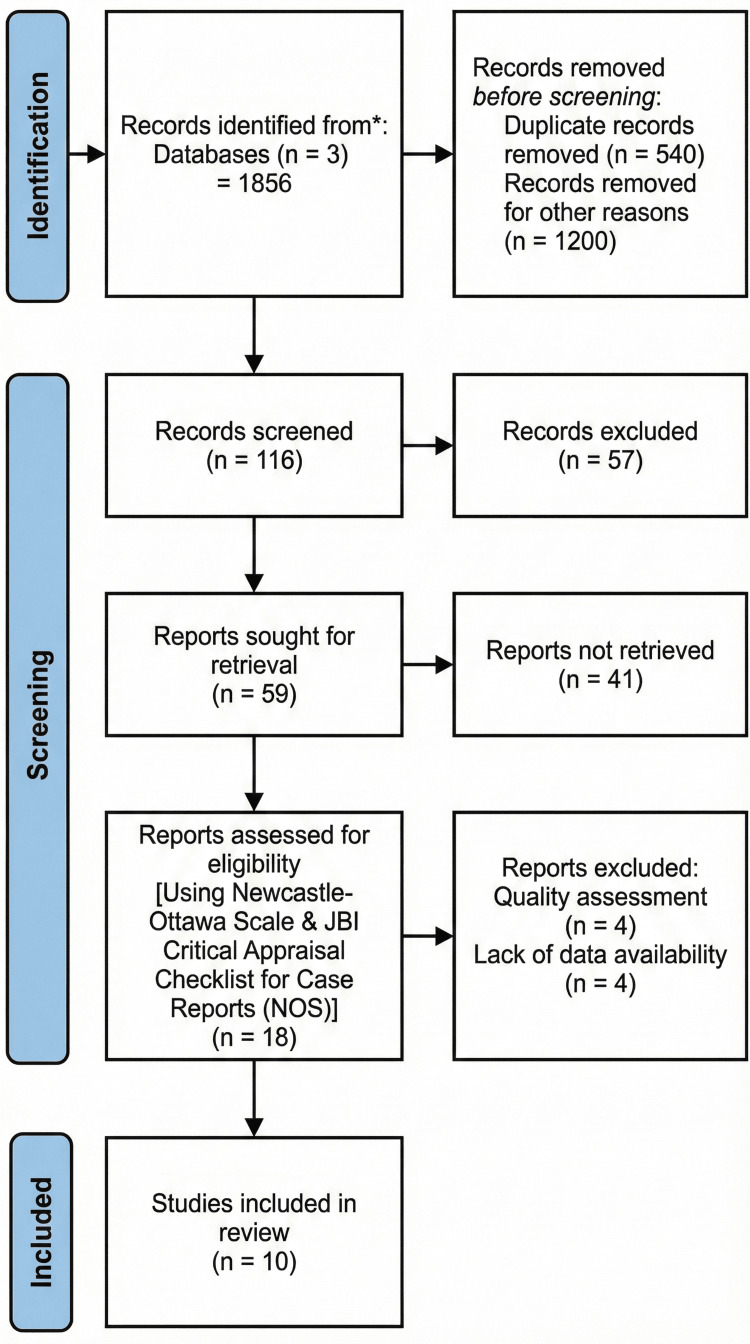
PRISMA flow diagram explaining study selection process. Credit: [9]

A comprehensive literature search conducted across PubMed, Scopus, and Google Scholar initially yielded 1,856 records. After title and abstract screening, 116 full-text articles were retrieved and assessed for eligibility. Of these, data extraction was completed for 59 studies. Quality appraisal using the NOS (for observational studies) and the JBI Critical Appraisal Checklist (for case reports) led to the exclusion of four studies (see Appendix 1). An additional four studies were excluded due to insufficient data; although corresponding authors were contacted for clarification, no responses were received. Finally, 10 studies that met all inclusion criteria and reported either antibiotic resistance profiles or biofilm production in *K. pneumoniae* isolates from India were included in the meta-analysis. These comprised six cross-sectional studies, three cohort studies, and one case report (Figure 1). The full search strategy, data extraction sheets, and author correspondence records are available in the OSF registry [8]. Table 1 provides the extracted data from the included studies.

**Table 1:** Full data extraction of all included studies. CRKP strong / Total strong and CRKP weak / Total weak refer to a stratified count of carbapenem-resistant *Klebsiella pneumoniae* (CRKP) isolates based on their biofilm-forming ability reported in studies. Studies included: [13-22]

Author, year	Study design	State, region	Study setting or sample source	Type of samples	Total number of isolates/*Klebsiella pneumoniae* isolates	CRKP strong	Total strong	CRKP weak	Total weak	Number of biofilm-positive isolates	Resistance genes identified
Filgona et al. (2015) [13]	Cross-Sectional	Uttar Pradesh	Hospital	Urine, blood, sputum, endotracheal tube, pus aspirates, intravascular catheter tip, ascitic fluid, wound swabs	238 *K. pneumoniae* isolates	~	~	~	~	~	~
Kammili et al. (2020) [14]	Cross-Sectional	Telangana	Hospital	Urine	1841/29	~	~	~	~	~	CTX-M-15, TEM-1, SHV-38, CTX-M-15+TEM-1
Bobbadi et al. (2021) [15]	Cross-Sectional	Andhra Pradesh	Diagnostic lab	Urine	504/85	~	~	~	~	15	*gyrA*, *rpoB*, *rmpA*, *uge*, *kfu*, aerobactin
Jaisal et al. (2024) [16]	Cross-Sectional	Uttar Pradesh	Rural hospital	Sputum, bronchoalveolar lavage, endotracheal aspirates, urine, blood, central line tips, umbilical catheter tips, wound swabs, pus	670/110	16	34	2	24	86	*bla*NDM, *bla*NDM + *bla*OXA-48, *bla*OXA-48
Desai et al. (2019) [17]	Cross-Sectional	Gujarat	Pathology labs	Urine	28 *K. pneumoniae*	~	~	~	~	28	~
Chatterjee et al. (2014) [18]	Cross-Sectional	New Delhi	Tertiary care hospital	Foley urinary catheters, double J (DJ) stents, urine	225/27	~	~	~	~	25	~
Singh et al. (2021) [19]	Cohort	Uttar Pradesh	Hospital ICU	Pus, blood, endotracheal aspirate, tissue, and sputum	22 *Klebsiella* isolates	11	11	3	3	22	*mcr-1*, *mgrB*, *bla*NDM, *bla*OXA-48, *bla*VIM, *bla*IMP, *bla*CTX-M, *bla*SHV, *bla*TEM, 16S rRNA methyltransferases (*armA*, *rmtB*, *rmtC*)
Devanga et al. (2020) [20]	Cohort	Tamil Nadu	Hospital ICU	Blood, endotracheal aspirates	72 *Klebsiella* isolates	18	20	15	22	50	*wcaG*, *magA*, *rmpA*, *rmpA2*, *wzc*, *wabG*, *treC* (capsular polysaccharide synthesis); *bcsA*, *pgaC* (adhesins); *luxS* (quorum sensing); aerobactin (*iutA*); allantoin (*allS*); type I and III fimbriae (*fimA*, *fimH*, *mrkD*); pili genes (*pilQ*, *ecpA*)
Mukherjee et al. (2023) [21]	Cohort	West Bengal	Hospital Neonatal ICU	Blood	285/107	~	~	~	~	28/12	*aac*(6′)-Ib, *bla*TEM, *bla*SHV, *bla*OXA, *bla*CTX-M-15, *bla*AmpC, *bla*NDM-1, *bla*NDM-5, *bla*OXA-232, *armA*, *rmtB*, *rmtC*, *qnrB1*, *qnrS1*, *oqxA*, *oqxB*, *aac*(6′)-Ib-cr
Paul et al. (2019) [22]	Case report	Kerala	Hospital	Blood	*Klebsiella pneumoniae* CRKP I	~	~	~	~	~	*aac*(6′)-Ib-cr, *armA*, *aadA2*, *aph*(3′)-V, *aac*(3)-IId, *baeR*, *bla*NDM-1, *bla*OXA-1, *bla*SHV-11, *bla*SHV-13, *bla*TEM-1A, *bla*CTX-M-15, *sul1*, *tetD*, *dfrA1*, *dfrA2*, *fosA*, *catB3*, *catA1*, *msr(E)*, *mph(D)*, *mph(E)*, *oqxA*, *oqxB*, *qnrB1*; mutations in ParC (S80I), PBP3 (S357N, D350N), EF-Tu (R234F), UhpT (E350Q), GyrA (D87G)

CRKP Prevalence and Biofilm Production in K. pneumoniae

Across the studies that quantified biofilm strength, a substantial proportion of isolates exhibited strong biofilm production. For example, Jaisal et al. [16] reported that 34/110 isolates were strong biofilm producers, with 16 of these demonstrating CRKP. In the same study, weak biofilm producers accounted for 24/110 isolates, with only two being CRKP. Devanga et al. [20] observed similar patterns, reporting 20 strong biofilm producers (18 CRKP) and 22 weak producers (15 CRKP) among 72 isolates. Singh et al. [19] found that all 11 strong biofilm-producing isolates from ICU patients were CRKP, compared to 3/3 weak producers. Although data were insufficient for formal pooled analysis, a consistent qualitative trend was observed: strong biofilm producers were more frequently carbapenem-resistant compared to weak producers. In studies with complete CRKP-biofilm stratification [16,19,20], the proportion of CRKP among strong producers ranged from 53% to 100%, while among weak producers, the proportion ranged from 8% to 100%, with lower rates generally observed in larger datasets.

Molecular characterization revealed the widespread presence of resistance determinants in biofilm-producing CRKP isolates. Carbapenemases (*bla*NDM, *bla*OXA-48, *bla*VIM, and *bla*IMP) were frequently detected, along with extended-spectrum β-lactamases (ESBLs) such as *bla*CTX-M, *bla*TEM, and *bla*SHV. Aminoglycoside resistance genes (*rmtB*, *armA*, and *rmtC*) and efflux pump regulators (*oqxA* and *oqxB*) were also common. Some isolates harbored multiple resistance determinants spanning up to 12 antibiotic classes, including mutations in target sites such as *ParC*, *PBP3*, and *GyrA*. The reviewed studies covered a wide geographic distribution across India, with isolates obtained from varied clinical sources, including urine, blood, respiratory samples (sputum, BAL, and endotracheal aspirates), wound swabs, catheter tips, and ascitic fluid. ICU settings (adult and neonatal) and device-associated infections (urinary catheters and DJ stents) were particularly prominent sources of CRKP strong biofilm producers, suggesting the role of invasive devices and critical-care environments as high-risk reservoirs. 

Distribution of Resistance Genes

**Figure 2:**
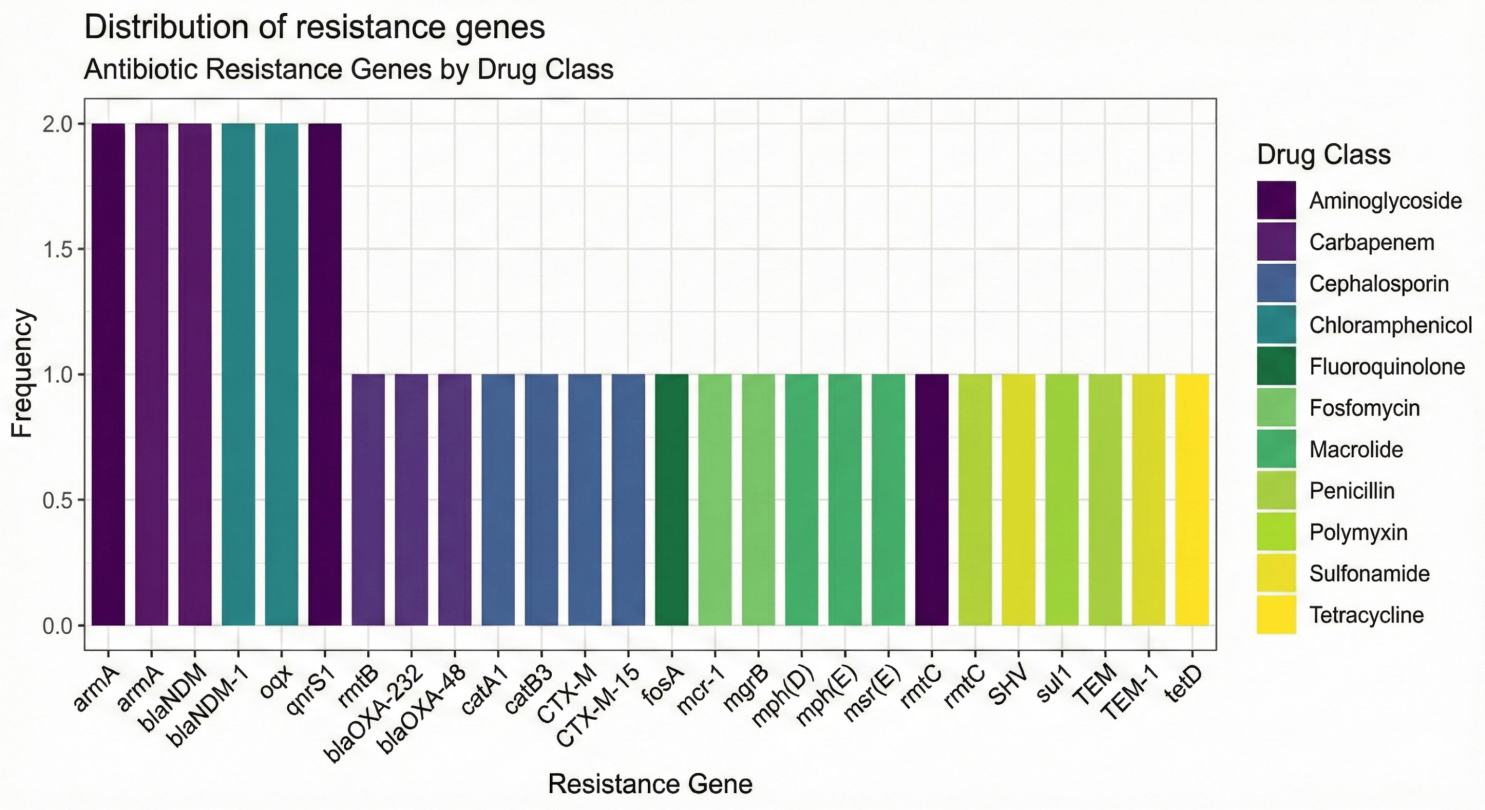
Barchart showing the distribution of antibiotic resistance genes by drug class in Klebsiella pneumoniae clinical isolates from India. Each cell represents the frequency with which a gene was reported across the included studies, stratified by its corresponding antibiotic class. Notably, genes encoding carbapenemases (e.g., *bla*NDM and *bla*OXA-48) and aminoglycoside-modifying enzymes were among the most frequently detected, pointing to the convergence of multidrug resistance mechanisms.

A total of 24 distinct antibiotic resistance genes were reported across the 10 included studies. The most frequently observed genes were *bla*NDM and its variants, followed by *qnrB1*, *oqxA*, *armA*, *rmtB*, *rmtC* (aminoglycoside and fluoroquinolone resistance), and ESBLs such as *CTX-M-15*, *TEM*, and *SHV* (Figure 2).

Genes were categorized by their associated antibiotic class; the frequency of these genes within each class is represented in the chart (Figure 2). Carbapenems, aminoglycosides, and penicillins exhibited the highest genetic diversity. Moreover, plasmid-mediated resistance genes such as *bla*OXA-48, *mcr-1*, and *mgrB* were also reported, indicating the potential for pan-drug resistance (PDR) in Indian clinical isolates. The aminoglycoside resistance genes *armA* (methyltransferase) and *rmtB* were each found in two studies, while *rmtC* appeared in one. These genes inhibit the binding of aminoglycosides to the 16S rRNA of the bacterial ribosome, effectively neutralizing bactericidal activity. Similarly, fluoroquinolone resistance was supported by *oqxA* (efflux pump) and *qnrB1* (DNA gyrase protection), which were both found in two studies, signifying widespread plasmid-mediated quinolone resistance (PMQR).

Additional β-lactamase genes, such as *CTX-M*, *TEM*, and *SHV*, contribute to broad-spectrum resistance by hydrolyzing third-generation cephalosporins and penicillins. These genes are often co-located with carbapenemases on the same plasmids, exacerbating the risk of MDR. Alarmingly, resistance to last-line agents like polymyxins was also observed via *mcr-1* and *mgrB*, each reported in separate studies. This raises concern about emerging PDR strains of *K. pneumoniae*. The distribution of resistance genes across multiple drug classes points to co-selection and horizontal gene transfer, especially within hospital environments. Genes related to macrolides (*mph*(D), *mph*(E), and *msr*(E)), chloramphenicol (*catA1* and *catB3*), sulfonamides (*sul1*), tetracyclines (*tetD*), and fosfomycin (*fosA*) were also identified, albeit less frequently, indicating the accumulation of resistance determinants across a wide antibiotic spectrum.

Post Hoc Meta-Analysis on Prevalence of blaNDM Gene

**Figure 3:**
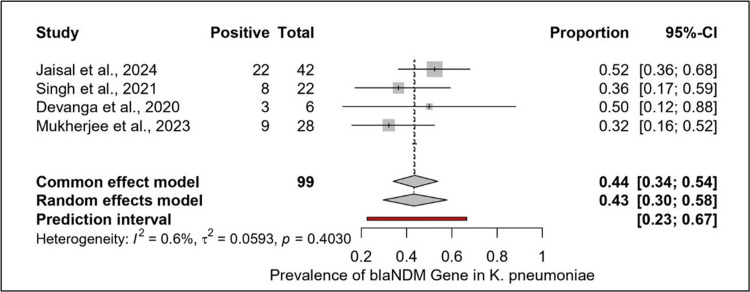
Forest plot showing the pooled prevalence of the blaNDM gene among clinical Klebsiella pneumoniae isolates in India. Four studies were included in the post hoc meta-analysis*. The pooled prevalence estimated by the random-effects model was 43% (95% CI: 30%-58%), with low heterogeneity (I² = 0.6%). The prediction interval (23%-67%) suggests the likely range of *bla*NDM prevalence across studies. * denotes that this post hoc meta-analysis was exploratory and hypothesis-generating. Studies included: [16,19-21]

**Figure 4:**
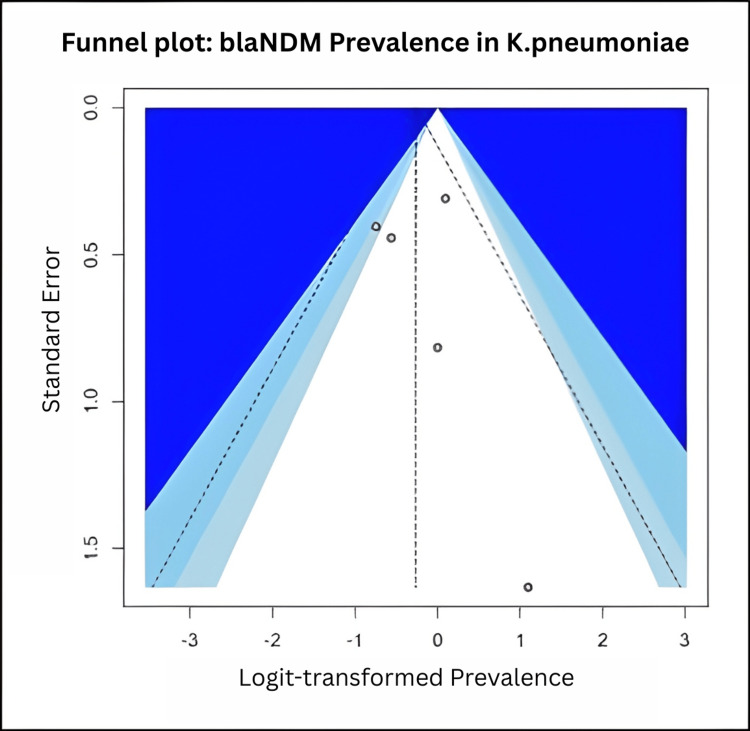
Funnel plot evaluating publication bias in the meta-analysis of blaNDM gene prevalence among Klebsiella pneumoniae isolates in India. Each circle represents a study, plotted by standard error (y-axis) and logit-transformed prevalence (x-axis). The vertical dashed line indicates the pooled estimate from the random-effects model. Contour shading corresponds to regions of statistical significance. The distribution appears visually symmetric, with no apparent missing studies in non-significant regions, suggesting no strong evidence of publication bias.

A post-hoc meta-analysis of *bla*NDM gene prevalence in Indian *K. pneumoniae* clinical isolates included four observational studies (n = 99 isolates; range 6-42 per study). The pooled prevalence estimate, using a random-effects model, was 0.43 (95% CI: 0.30-0.58), with a 95% prediction interval of 0.23-0.67. Heterogeneity was negligible (I² = 0.6%, τ² = 0.0593, p = 0.403). Leave-one-out sensitivity analysis did not materially change the pooled estimate (range 0.41-0.45). Given the low heterogeneity, results were consistent across the included studies. This analysis underscores a persistently high prevalence of *bla*NDM among *K. pneumoniae* in India (Figures 3-4).

Discussion

The results of this systematic review provide a comprehensive evaluation of the association between biofilm-forming ability and CRKP clinical isolates in India. In addition, we synthesize the prevalence of the *bla*NDM gene and map the resistome profile across various antibiotic classes. Given the increasing burden of healthcare-associated infections (HAIs) and AMR, particularly in low- and middle-income countries, our findings hold important clinical and public health implications.

Although case reports generally represent a lower level of evidence and are often excluded from systematic reviews, one case report was retained in the present review due to its relevance to the study objectives. The review specifically focused on *K. pneumoniae* infections reported from India, and this case report provided a detailed molecular characterization of resistance determinants in a clinical isolate, including the identification of specific resistance genes. As such, it offered valuable descriptive insight into the genetic mechanisms underlying AMR within the regional context. However, recognizing the inherent methodological limitations of case reports, this study was included only for qualitative discussion and was excluded from quantitative synthesis and prevalence estimation to avoid influencing the statistical interpretation of the findings.

Biofilms are complex microbial communities embedded within an extracellular polymeric matrix, providing structural protection and facilitating the survival of bacteria under hostile conditions, including antibiotic pressure [1]. In our review, most eligible studies reported a higher proportion of CRKP among strong biofilm producers compared to weak or non-biofilm producers. Although the association was consistent in direction, the limited number of studies and variability in biofilm detection methods precluded a formal meta-analysis. This association should, therefore, be considered hypothesis-generating rather than confirmatory.

International evidence presents a heterogeneous picture. Rahdar et al. [23] reported that 77.9% of CRKP isolates were strong biofilm formers, with a statistically significant association. In contrast, Cusumano et al. [24] observed that CRKP strains were less likely to form strong biofilms (OR: 0.09; 95% CI: 0.02-0.33), suggesting a possible fitness trade-off in certain genetic backgrounds. Such conflicting patterns highlight the potential influence of geographical, genetic, and clinical factors on the biofilm-resistance relationship and reinforce the need for region-specific research. Global surveillance programs have highlighted the increasing burden of AMR worldwide. Data from the WHO Global Antimicrobial Resistance and Use Surveillance System (GLASS) indicate rising resistance trends across multiple pathogens, including *K. pneumoniae*, reported from more than 100 participating countries [25]. Similarly, the CDC Antibiotic Resistance Threats report estimates that antimicrobial-resistant infections cause more than 2.8 million infections and over 35,000 deaths annually in the United States, emphasizing the substantial clinical and public health impact of AMR [26]. Our focused synthesis of antibiotic-resistant *K. pneumoniae* clinical isolates from India reflects a comparable pattern of escalating resistance burden. 

While our qualitative synthesis of Indian *K. pneumoniae* clinical isolates reported a positive association between strong biofilm formation and carbapenem resistance, a U.S.-based study by Cusumano et al. [24] found the opposite trend. Their analysis of 139 genetically diverse isolates and hospital strains revealed that CRKP were less likely to be strong biofilm formers (OR: 0.09; 95% CI: 0.02-0.33). This inverse correlation led the authors to propose a possible fitness trade-off in CRKP, in which biofilm formation may be attenuated to conserve energy for resistance expression and survival under antimicrobial pressure. In contrast, our qualitative summary from Indian isolates suggests that CRKP strains were more likely to form strong biofilms. This discrepancy highlights the heterogeneity of resistance-biofilm relationships across geographic and genetic backgrounds [24].

Although not prespecified in our protocol, we conducted a post hoc meta-analysis of the prevalence of the *bla*NDM gene, a clinically critical carbapenemase. This decision was guided by the frequent reporting of *bla*NDM in our included studies and its epidemiological importance in India. The pooled prevalence was notably high, suggesting widespread dissemination of *bla*NDM-positive *K. pneumoniae* in Indian clinical settings. The *bla*NDM gene encodes a metallo-β-lactamase that hydrolyzes almost all β-lactams, including carbapenems, leaving few therapeutic options. The dissemination of *bla*NDM is particularly concerning due to its localization on mobile genetic elements, enabling interspecies and intraspecies transfer [27]. A prior systematic review and meta-analysis by Dadashi et al. [27], which examined studies between 2005 and 2016 across various Asian countries, estimated a pooled prevalence of 32.5% (95% CI: 22.6-44.3) for *bla*NDM-producing *K. pneumoniae*. While their analysis provided valuable insights into the regional burden of NDM-positive strains, our updated synthesis focuses exclusively on Indian clinical isolates, incorporates more recent studies up to 2024, and presents a notably higher pooled prevalence of 43% (95% CI: 30%-58%). This reflects both the temporal evolution and local intensification of carbapenem resistance mechanisms within Indian healthcare settings.

Our focus was exclusively on Indian studies to offer more granular detail on NDM prevalence trends within a high-burden country, allowing for stronger policy and clinical relevance to Indian infection control frameworks. Moreover, while the prior review noted a rise in NDM prevalence post-2010, our findings confirm that this trend has not only persisted but has possibly intensified, especially in ICU and nosocomial infection settings.

Another contribution of our study lies in the qualitative mapping of the resistome across *K. pneumoniae* isolates. We identified 24 unique antibiotic resistance genes, with 43 cumulative gene occurrences spanning 12 drug classes, and a high frequency of genes associated with aminoglycosides (*rmtB* and *armA*), β-lactams (*bla*NDM, *bla*CTX-M, and *bla*OXA), and fluoroquinolones (*qnrB* and *aac(6′)-Ib-cr*). The presence of multiple resistance determinants within single isolates, such as those reported by Paul et al. [22] and Devanga et al. [20], indicates the evolution of XDR phenotypes in hospital-acquired *K. pneumoniae* infections. The gene bank data of *K. pneumoniae* (16S PCR and full genome) and antibiotic resistance genes reported in identified studies are available in the supplementary files and OSF database. The resistome gene frequency analysis demonstrated that *bla*NDM remains the most dominant gene across studies, followed by *bla*OXA-48, *bla*CTX-M-15, and aminoglycoside-modifying enzymes. Notably, resistance genes to polymyxins (*mgrB* and *mcr-1*) were detected in some isolates, highlighting the erosion of last-line treatment options. 

The current review combines qualitative synthesis of the biofilm-CRKP association with a meta-analysis of *bla*NDM prevalence and detailed resistome mapping specific to India, to help guide future research efforts in this direction - specifically, studies to be conducted on *K. pneumoniae* in India. We have documented the entire study process, including protocols, codes, data extraction, author correspondence, and sequences reported in included studies, in the OSF database, which is freely accessible to everyone [8]. Although we adhered to rigorous scientific standards and followed PRISMA guidelines in conducting and reporting this systematic review and meta-analysis, certain limitations in the current approach need to be acknowledged. The small number of studies on the biofilm-CRKP association limited us to qualitative synthesis, reducing the ability to draw strong causal inferences. Heterogeneity in phenotypic biofilm assays (e.g., microtiter plate vs. Congo red agar) and molecular detection methods could affect comparability. Additionally, some included studies were small or case-based, potentially skewing resistance gene frequency estimates.

The findings of this study have critical applications for clinicians, microbiologists, and policymakers. Future research should aim to standardize biofilm detection protocols and incorporate biofilm strength into surveillance databases. Large-scale genomic epidemiology studies can further elucidate the co-localization of biofilm and resistance genes. Additionally, national networks could be established to report real-time data on gene prevalence, enabling dynamic risk stratification and antimicrobial policy formulation.

## Conclusions

The current synthesis identifies a consistent qualitative trend toward higher carbapenem resistance among strong biofilm-forming *K. pneumoniae* clinical isolates reported from India. While causality cannot be inferred from the available evidence, the observed pattern suggests a potential interaction between biofilm-associated virulence traits and AMR mechanisms, warranting further large-scale, methodologically standardized investigations. The pooled prevalence of the *bla*NDM gene (43%) highlights the widespread dissemination of metallo-β-lactamase-producing strains in Indian healthcare settings. In addition, qualitative resistome mapping demonstrated the accumulation of resistance determinants across multiple antibiotic classes, including genes conferring resistance to last-line agents, such as polymyxins and aminoglycosides, particularly in ICU and hospital-associated infections.

These findings underscore the importance of integrating molecular resistance gene surveillance and standardized biofilm assessment into national AMR monitoring frameworks. Strengthened infection prevention and control strategies, alongside robust antimicrobial stewardship programs, will be essential to mitigate the continued spread of high-risk MDR *K. pneumoniae* clones in India. This evidence synthesis provides context-specific insights to inform future research priorities and support clinical and public health decision-making.

## Appendices

Appendix 1

**Table 2:** Quality assessment table using the JBI Critical Appraisal Checklist for case report. Study included: [22]

Study	Patient’s demographic characteristics clearly described? (Y, N, UC)	Was the patient’s history clearly described and presented as a timeline?	Was the current clinical condition of the patient on presentation clearly described?	Were diagnostic tests or assessment methods and the results clearly described?	Were the intervention(s) or treatment procedure(s) clearly described?/Was the post-intervention clinical condition clearly described?	Were adverse events (harms) or unanticipated events identified and described?	Does the case report provide takeaway lessons?	Decision
Paul et al. (2019)	Y	Y	Y	Y	N	N	Y	Include

**Table 3:** Quality assessment table using the Newcastle-Ottawa scale for cross-sectional study designs. Studies included: [13-18]

Study title	Rate the representativeness of the sample, sample size, and response rate. A maximum of 5 stars can be awarded.	Evaluate whether the study accounted for confounding factors. Provide up to 2 stars based on the methods used to adjust for these variables.	Assess the validity and reliability of the outcome measurement. Include considerations like consistency and follow-up where applicable. Assign up to 3 stars.	Add the scores from all three domains. The maximum score is 10 stars.	Assign a rating based on the total stars: Poor (0-3), Fair (4-6), Good (7-8), Very Good (9-10).
Filgona et al. (2015)	3	1	3	7	Good
Kammili et al. (2020)	4	2	3	9	Very Good
Bobbadi et al. (2021)	3	1	4	8	Good
Jaisal et al. (2024)	3	2	2	6	Good
Desai et al. (2019)	3	1	2.5	~7	Good
Chatterjee et al. (2014)	3	1	4	8	Very Good

**Table 4:** Quality assessment table using the Newcastle-Ottawa scale for cohort study designs. Studies included: [19-21]

Study title	Selection (4 stars max) 1. Representativeness of the exposed cohort 2. Selection of the non-exposed cohort (control group) 3. Ascertainment of exposure 4. Demonstration that the outcome of interest was not present at study start	Comparability (2 stars max) 1. Comparability of groups based on design or analysis 2. Additional adjustments for other confounders	Outcome (3 stars max) 1. Outcome 2. Sufficient follow-up duration 3. Adequacy of follow-up.	Decision
Singh et al. (2021)	3	1	3	Include
Devanga et al. (2020)	3	2	3	Include
Mukherjee et al. (2023)	3	1	3	Include

**Table 5:** Records of Klebsiella pneumoniae genomic sequences submitted to GenBank from various studies included in systematic review. Studies included: [14,19,22]

Study	Sequence details	Accession number	NCBI link
Kammili et al. (2020)	*Klebsiella pneumoniae* strain ESBL/SHV/GMC/TG/382 SHV family beta-lactamase (*bla*SHV) gene, partial cds	MH745712	https://www.ncbi.nlm.nih.gov/nuccore/MH745712
Kammili et al. (2020)	*Klebsiella pneumoniae* strain ESBL/SHV/GMC/TG/260 SHV family beta-lactamase (*bla*SHV) gene, partial cds	MH745711	https://www.ncbi.nlm.nih.gov/nuccore/MH745711
Kammili et al. (2020)	*Klebsiella pneumoniae* strain ESBL/SHV/GMC/TG/1583 SHV family beta-lactamase (*bla*SHV) gene, partial cds	MH745713	https://www.ncbi.nlm.nih.gov/nuccore/MH745713
Paul et al. (2019)	*Klebsiella* sp. strain CRKP I 16S ribosomal RNA gene, partial sequence	MK640671	https://www.ncbi.nlm.nih.gov/nuccore/MK640671
Singh et al. (2021)	*Klebsiella pneumoniae* strain Kp1 MCR family phosphoethanolamine--lipid A transferase (mcr) gene, partial cds	MN652072	https://www.ncbi.nlm.nih.gov/nuccore/MN652072
Singh et al. (2021)	*Klebsiella pneumoniae* strain Kp19 MCR family phosphoethanolamine--lipid A transferase (mcr) gene, partial cds	MN652090	https://www.ncbi.nlm.nih.gov/nuccore/MN652090
Singh et al. (2021)	*Klebsiella pneumoniae* strain CRkp20 insertion sequence IS-1, complete sequence; and *mgrB* gene, partial sequence	MW389562	https://www.ncbi.nlm.nih.gov/nuccore/MW389562
Singh et al. (2021)	*Klebsiella pneumoniae* strain CRkp22 insertion sequence IS-5, complete sequence; and *mgrB *gene, partial sequence	MW389564	https://www.ncbi.nlm.nih.gov/nuccore/MW389564
